# Newborn Screening for Primary Congenital Hypothyroidism: Estimating Test Performance at Different TSH Thresholds

**DOI:** 10.1210/jc.2018-00658

**Published:** 2018-08-02

**Authors:** Rachel L Knowles, Juliet Oerton, Timothy Cheetham, Gary Butler, Christine Cavanagh, Lesley Tetlow, Carol Dezateux

**Affiliations:** 1 Life Course Epidemiology and Biostatistics, UCL Great Ormond Street Institute of Child Health, London, United Kingdom; 2 Newcastle University and Department of Paediatric Endocrinology, Royal Victoria Infirmary, Newcastle-upon-Tyne, United Kingdom; 3 Paediatric and Adolescent Endocrinology, University College London Hospitals NHS Foundation Trust and UCL Great Ormond Street Institute of Child Health, London, United Kingdom; 4 National Newborn Blood Spot Screening Programme, Public Health England, London, United Kingdom; 5 Department of Clinical Biochemistry, Manchester University NHS Foundation Trust, Manchester, United Kingdom; 6 Centre for Primary Care and Public Health, Barts and the London School of Medicine and Dentistry, Queen Mary University London, United Kingdom

## Abstract

**Context:**

Active surveillance of primary congenital hypothyroidism (CH) in a multiethnic population with established newborn bloodspot screening.

**Objective:**

To estimate performance of newborn screening for CH at different test thresholds and calculate incidence of primary CH.

**Design:**

Prospective surveillance from June 2011 to June 2012 with 3-year follow-up of outcomes. Relative likelihood ratios (rLRs) estimated to compare bloodspot TSH test thresholds of 6 mU/L and 8 mU/L, with the nationally recommended standard of 10 mU/L for a presumptive positive result.

**Setting:**

UK National Health Service.

**Patients:**

Clinician notification of children aged <5 years investigated following clinical presentation or presumptive positive screening result.

**Main Outcome Measure(s):**

Permanent primary CH status determined by clinician report of continuing T4 requirement at 3-year follow-up.

**Results:**

A total of 629 newborns (58.3% girls; 58.7% white ethnicity) were investigated following presumptive positive screening result and 21 children (52.4% girls; 52.4% white) after clinical presentation; 432 remained on treatment at 3-year follow-up. Permanent CH incidence was 5.3 (95% CI, 4.8 to 5.8) per 10,000 infants. With use of locally applied thresholds, sensitivity, specificity, and positive predictive value were 96.76%, 99.97%, and 66.88%, respectively. Compared with a TSH threshold of 10 mU/L, positive rLRs for 8 mU/L and 6 mU/L were 1.20 (95% CI, 0.82 to 1.75) and 0.52 (95% CI, 0.38 to 0.72), and negative rLRs were 0.11 (95% CI, 0.03 to 0.36) and 0.11 (95% CI, 0.06 to 0.20), respectively.

**Conclusions:**

Screening program performance is good, but a TSH threshold of 8 mU/L appears superior to the current national standard (10 mU/L) and requires further evaluation. Further research should explore the implications of transient CH for screening policy.

## Teaser

Newborn bloodspot screening for primary congenital hypothyroidism was assessed at different test thresholds. Lowering the national threshold should improve performance of the UK screening program.

Primary congenital hypothyroidism (CH) affects ∼1 in 2000 children born in the United Kingdom each year. It is estimated that 8% to 28% of children presenting clinically will develop severe intellectual disability, defined as an IQ <70 (1). Newborn screening to identify those with CH enables timely T4 replacement therapy and potentially prevents or mitigates this disability (2), Newborn screening was introduced in the United Kingdom in 1981 and is currently based on whole-blood TSH concentrations measured in dried bloodspots collected 5 days postnatally (3). Secular increases in the proportion of babies with presumptive positive screening results (1) may reflect many factors, including increasing ethnic diversity (4); changes in maternal iodine status (5, 6); and reduction over time in the lower limit of TSH threshold used to define a presumptive positive result, reflecting technological advances in laboratory measurement (1, 7).

The UK national standards recommend confirmatory diagnostic testing in all infants with screening bloodspot TSH (whole blood) ≥20 mU/L, or ≥10 mU/L after repeat testing for borderline results (Table 1); in practice, however, TSH thresholds vary for technological and historical reasons (1), and the current performance of the national program has not been appraised. At the time of this study, 12 of the 16 UK newborn screening laboratories used a TSH threshold below the recommended national standard, largely because of concerns about false-negative results. This provided a rare opportunity to evaluate screening test performance at different TSH thresholds within an existing national program involving a multiethnic population of 700,000 births per year.

**Table 1.** Screening and Surveillance Definitions

UK National Guidelines for Newborn Blood Spot Screening
The first newborn bloodspot sample is taken at 5 d of age in all babies.
Babies born at <32 wk gestation also have a second (repeat) blood spot sample at 28 d of age or on the day of discharge home, whichever is sooner, as immaturity may mask CH.
A presumptive positive screening result requiring referral for diagnostic investigation is defined as a TSH concentration of >20 mU/L on the newborn blood spot (whole blood) sample; a concentration between 10 and 20 mU/L is a "borderline" result requiring a repeat screen and diagnostic referral if the TSH level remains ≥10 mU/L in the second blood spot sample.
Clinical referral guidelines recommend thyroid function tests (serum TSH and free T4) to confirm the diagnosis after a presumptive positive screen as well as ultrasonography and/or radio-isotope scanning to determine the underlying thyroid gland abnormality (5).
Treatment: oral T4, which should be initiated by 21 d of age (2, 5).
Reporting case definition

Reporting Case Definition for the Surveillance Study
Any child age ≤5 y of age who, during the past month, has:
• Been referred for diagnostic confirmation following a newborn screening test result suggestive of primary CH OR
• Has been confirmed with a diagnosis of primary CH (known or considered likely to be present from birth), based on a serum TSH ≥ 10 mU/L.

We carried out a prospective United Kingdom–wide active surveillance study to identify all confirmed diagnoses of primary CH in children age <5 years, regardless of screening results. Because CH may be transient in the early years, we obtained reports of outcomes in notified infants after diagnosis and, with expert advice from pediatric endocrinologists, developed and applied standardized criteria for defining confirmed and probable CH status from clinician reports at 3-year follow-up. To inform future screening policy, we assessed incremental changes in the detection rate, false-positive rate, and likelihood ratio of two alternatives to the current recommended threshold of ≥10 mU/L for defining a presumptive positive screening result (≥8 mU/L or ≥6 mU/L).

## Materials and Methods

### Ascertainment of cases

Children with newly diagnosed CH were identified by active surveillance through the British Pediatric Surveillance Unit national clinical pediatric surveillance system and through a concurrent laboratory reporting system, involving all 16 UK newborn screening laboratories. Each month, secondary and tertiary care pediatricians and laboratory directors notified all children meeting the reporting case definition (Table 1), and full clinical details were obtained from the notifying clinician and laboratory using online questionnaires (8). Laboratory and clinician notifications were matched by using birth date, National Health Service number or equivalent, sex, and postal code district. If a case had not been reported by both sources, we asked pediatricians or laboratories to complete a questionnaire or provide further clinical details.

### Follow-up and outcome adjudication

All children were followed up annually by using online or postal questionnaires sent to clinicians until one of the following endpoints occurred: completion of 3 years of follow-up, death, confirmation of CH, and discharge from clinician care or loss to follow-up. Collected data included details of screening and diagnostic test results, clinical presentation, and management.

An independent expert panel, comprising two pediatric endocrinologists and one screening laboratory director, reviewed every child’s deidentified data to determine (1) eligibility for study inclusion and (2) outcome at 3 years. Children had confirmed permanent CH at 3 years if a persisting requirement for T4 was confirmed by a trial off therapy (withdrawal of T4 replacement therapy and re-evaluation of thyroid function tests to confirm or exclude CH), or radioisotope or ultrasonography scan results confirming thyroid agenesis or ectopic thyroid, or continuing requirement for high-dose levothyroxine (≥50 μg per day) indicated by regular review of thyroid function by pediatricians. Children had “probably permanent” CH if confirmation of CH as defined above was absent but the clinician was continuing levothyroxine at final follow-up. Children were confirmed as not CH if not receiving treatment by 3-year follow-up; children who had a period on levothyroxine before treatment was discontinued, following a trial off therapy or other clinical evaluation (not specified by the clinician), were defined as having transient CH.

### Test performance, incidence, and standardized population

Incidence of permanent CH diagnosis in UK infants was estimated by using monthly live birth data from the Office for National Statistics (9), National Registrations Scotland (10), and Northern Ireland Registration and Statistics Authority, for England and Wales, Scotland, and Northern Ireland, respectively (11). Ethnic groupings were white, Asian, black, mixed, and other (UK Census 2011 categories) (12). Incidence rates by sex, gestation, and ethnicity were estimated for England only by using Office for National Statistics live birth data [n = 693,748 live births (13)]; incidence rate ratios (IRRs) were estimated for comparison with reference categories (Table 2). Analyses comparing screen thresholds used standardized English live birth data (13), adjusted for between-laboratory population differences by sex, gestation, and ethnicity that could influence screening outcomes (4).

**Table 2.** Annual Incidence of Diagnosis of CH per 10,000 Live Births in England

Variable	Confirmed/Probable CH (n)	Births in England (n)^*a*^	Incidence per 10,000 Live Births (95% CI)	Rate Ratio ^*b*^ (95% CI)
Sex				
Male	148	338,081	4.4 (3.7–5.1)	Reference
Female	227	355,667	6.4 (5.6–7.3)	1.5 (1.2–1.8)
Not known	0	2592	—	—
Gestation at birth				
≥32 wk	354	683,829	5.2 (4.7–5.7)	Reference
<32 wk	19	9919	19.2 (11.5–29.9)	3.7 (2.2–5.9)
Not known	2	2592	—	—
Ethnicity				
White	231	510,586	4.5 (4.0–5.1)	Reference
Asian	82	73,466	11.2 (8.9–13.9)	2.5 (1.9–3.2)
Black	6	36,264	1.7 (0.6–3.6)	0.4 (0.1–0.8)
Mixed	23	34,969	6.6 (4.2–9.9)	1.5 (0.9–2.2)
Chinese	7	3,724	18.8 (7.6–38.7)	4.2 (1.7–8.7)
Other	12	13,484	8.9 (4.6–15.5)	2.0 (1.0–3.5)
Not known	14	23,847	—	—

^a^ Denominators are from 693,748 live births in England by sex, ethnicity, and gestation between July 2011 and June 2012 (data provided by Professor M. Cortina-Borja); the numerator is 375 probable/confirmed CH cases in England only (these denominators were not available for Scotland, Northern Ireland, and Wales).

^b^ The IRR is estimated for the incidence rate within each category compared with the reference.

Performance of the UK newborn screening program in detecting confirmed/probably permanent CH was evaluated from 2011 to 2012 (n = 813,087 live births), after exclusion of four infants diagnosed before screening and four with indeterminate outcome. In separate sensitivity analyses, children with probably permanent CH were assigned to the not CH category and infants with indeterminate outcome were assumed to be all true-positive or all false-positive cases.

Laboratories reported actual TSH values for positive screen results, and all values below the local threshold, as screen negative; therefore, a continuous receiver-operating characteristic curve could not be plotted to compare thresholds. Instead, three groups of English screening laboratories were defined by the lower TSH threshold each used: group 1 (n = 5 laboratories; TSH ≥5 mU/L or ≥6 mU/L), group 2 (n = 3; TSH ≥8 mU/L), and group 3 (n = 4; TSH ≥10 mU/L), and screening performance compared between groups. Because populations served by the laboratories differed in ethnic preterm birth rate profiles, we directly standardized populations (14) for comparison. We applied screen-positive rates by sex, ethnicity, and gestation from each laboratory group to the English population of 693,748 live births and adjusted the results to a population of 100,000 infants. The tradeoff between sensitivity and specificity for each laboratory group was plotted on a receiver-operating characteristic curve of sensitivity vs false-positive rate (1 − specificity).

Test performance at different TSH thresholds was compared by estimating positive (rLR+) and negative (rLR−) relative likelihood ratios using the method described by Hayen *et al.* (15) and assuming that a threshold of TSH ≥6 mU/L and TSH ≥8 mU/L were replacement screening thresholds for TSH ≥10 mU/L. Where the rLR+ for the new threshold is > 1 compared with the current threshold, this indicates that the new threshold is more likely to correctly assign a positive screen result to a child with CH, whereas an rLR− for the new threshold of <1 indicates the new threshold is less likely to incorrectly assign a positive screen result to a child without CH.

### Statistical analysis

Statistical analyses were performed by using Stata SE13 (Stata Corp., College Station, TX). Research ethics approval (Cambridge South REC; 11/EE/0152) and Section 251 support for the study were obtained (ECC 3-04(k)/2011).

## Results

There were 518 notifications from clinicians and 704 from laboratories. We excluded 75 duplicates (cases reported twice through the same source), 118 cases diagnosed before 1 July 2011, or not meeting our case definition (including non-UK births), and 41 cases that remained unverified because sufficient clinical details were not provided. Of those remaining, 338 were matched notifications reported by both laboratory and clinician sources.

All further analyses are based on 650 individual cases reported to the study during the 12 months between 1 July 2011, and 30 June 2012, comprising 629 children investigated after a presumptive positive newborn screen result and 21 reported as clinically detected by pediatricians. The total population screened was 813,087 (Table 3), of which 1.5% of babies were born at <32 weeks’ gestation and followed the preterm screening pathway, which included a repeat whole blood sample (Table 1).

**Table 3.** Performance of UK Newborn Screening Program for CH, 2011 to 2012

Variable	Confirmed/Probable CH (n)	CH Excluded (n)	Total (n)
Screen positive^*a*^	418	207	625
Screen negative^*a*^	14	812,448	812,462
Total	432	812,655	813,087

^a^ Screen result as defined by local laboratory TSH thresholds; outcome as defined at 3-y follow-up.

Children reported after a presumptive positive screen result were more likely to be girls (n = 367; 58.3%) and of white (n = 369; 58.6%) or Asian (n = 128; 20.3%) ethnicity. Fifty (7.9%) babies were born at <32 weeks’ gestation. Twelve children died, and all deaths were associated with prematurity or comorbidities; 1 infant was being treated for CH, 10 did not have CH, and 1 died before diagnostic tests were completed.

Of 21 clinically detected children,11 were girls, 11 were of white ethnicity, and 6 were born at <32 weeks’ gestation; one death occurred, which was unrelated to CH. CH was not suspected at newborn screening in 17 (screen negative) of these children and 4 were referred for investigation before the screening results were available; we refer to all of these as clinically detected cases because they were not identified through the newborn screening pathway.

### Diagnostic outcomes

#### Infants with a presumptive positive screen

**Figure 1.**
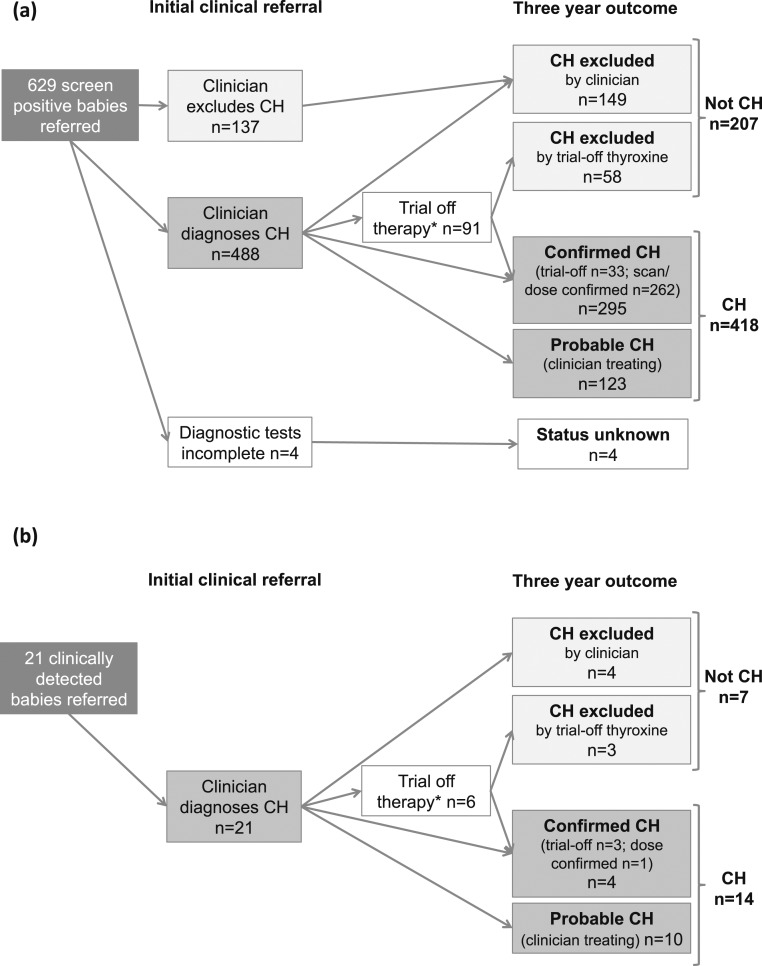
Flow diagram of outcomes at initial clinical referral and 3-y follow-up. (a) For 629 babies referred as screen positive. (b) For 21 babies referred as clinically detected.

At initial clinical referral, 488 (77.6%) of 629 children were diagnosed with CH and commenced levothyroxine; CH was excluded in 137 (21.8%) infants [Fig. 1(a)]. Diagnostic tests remained incomplete in four children (indeterminate outcome), one of whom died.

By 3 years of age, 295 children had confirmed permanent CH, of whom 33 had a trial off therapy, 165 had scan confirmation of agenesis or ectopic thyroid, and 97 required high-dose levothyroxine. A further 123 children had probably permanent CH. CH was excluded in 207 children [trial off therapy (n = 58) or other clinical evaluation (n = 149); Fig. 1(a)], of whom 70 received thyroxine for <3 years (transient CH). Of 50 screen-positive babies born at <32 weeks’ gestation, 16 had confirmed/probably permanent CH at 3 years.

#### Clinically detected children

At initial clinical referral, 20 of 21 children were diagnosed with CH and started levothyroxine; CH was excluded before treatment in one (Supplemental Table 1). Six children were born at <32 weeks’ gestation and had a repeat screen, and five were born at between 32 weeks’ and <37 weeks’ gestation. Four children suspected before screening had comorbidities and/or family history, and all remained on treatment at 3 years. Two of these babies had a blood spot TSH ≥ 10 mU/L screening (20 and 40 mU/L); however, they were referred before these screening results were reported.

By 3-year follow-up, four children had confirmed permanent CH; three of these had a trial off therapy and one required high-dose thyroxine [Fig. 1(b)]. These children presented with a congenital anomaly, family history, or prolonged jaundice; all had bloodspot TSH < 8 mU/L and started levothyroxine by age 3 months. Ten children had probably permanent CH at 3 years; all bloodspot TSH were ≤8 mU/L (and <6 mU/L in seven children). Four of six babies born at <32 weeks’ gestation had confirmed/probably permanent CH at 3 years. CH was excluded in seven children by 3 years; six had transient CH [confirmed by trial off therapy (n = 3) or other clinical evaluation (n = 3)] and one never started treatment.

### Incidence of CH

There were 432 infants born between 1 July 2011, and 30 June 2012, and were subsequently diagnosed with confirmed/probably permanent CH: 418 with a positive screen [Fig. 1(a)] and 14 clinically detected [Fig. 1(b)]. No child presenting after age 1 year had confirmed/probably permanent CH. UK birth prevalence was 5.3 (95% CI, 4.8 to 5.8) per 10,000 live births.

Incidence of permanent CH by sex, gestation, and ethnicity was estimated for English live births (Table 2). Incidence of permanent CH was significantly higher for girls [IRR, 1.5 (95% CI, 1.2 to 1.8)], and for infants born before 32 weeks’ gestation compared with those born at or after 32 weeks [IRR, 3.7 (95% CI, 2.2 to 5.9)]. Compared with children of white ethnicity [IR, 4.5 (95% CI, 4.0 to 5.1)], children of Asian and Chinese ethnicity had a significantly higher incidence of permanent CH [IRR, Asian: 2.5 (95% CI, 1.9 to 3.2); Chinese: 4.2 (95% CI, 1.7 to 8.7)], whereas children of black ethnicity had a lower incidence [IRR, 0.4 (95% CI, 0.1 to 0.8)].

### Screening program performance

Evaluation of United Kingdom–wide screening program performance, using locally determined TSH thresholds, demonstrated a high sensitivity of 96.76% (95% CI, 94.62% to 98.22%) and specificity of 99.97% (95% CI, 99.97% to 99.98%), for a positive predictive value (PPV) of 66.88% (95% CI, 63.04% to 70.56%) (Table 4). The likelihood ratio for a positive screen result, or the odds of a child having permanent CH if the screening test result is positive, was high at 3799. Sensitivity analyses assigning children with probable CH to "not CH" demonstrated similar screening sensitivity [95.47% (95% CI, 92.54% to 97.28%)] and specificity [99.96% [95% CI, 99.95% to 99.96%)] but lower PPV [47.20% (95% CI, 43.32% to 51.12%)]. Sensitivity analyses reassigning infants with indeterminate outcomes did not significantly alter test performance (data not shown).

**Table 4.** Screening Performance

Test Characteristic	Value (95% CI), %
Sensitivity	96.76 (94.62, 98.22)
Specificity	99.97 (99.97, 99.98)
PPV	66.88 (63.04, 70.56)
False-positive rate	0.03 (0.02, 0.03)
LR+	3799
LR−	0.03

LR, likelihood ratio.

### Screening performance at different bloodspot TSH thresholds

Screening performance at three TSH thresholds used by different groups of English laboratories (≥6 mU/L, ≥8 mU/L, ≥10 mU/L) was compared for a population of 100,000 English live births standardized by sex, gestation, and ethnicity (Supplemental Table 2). At TSH thresholds lower than the national standard (≥10 mU/L), the sensitivity and false-positive rate increased, and PPV decreased, being 62.2% at ≥6 mU/L.

**Figure 2.**
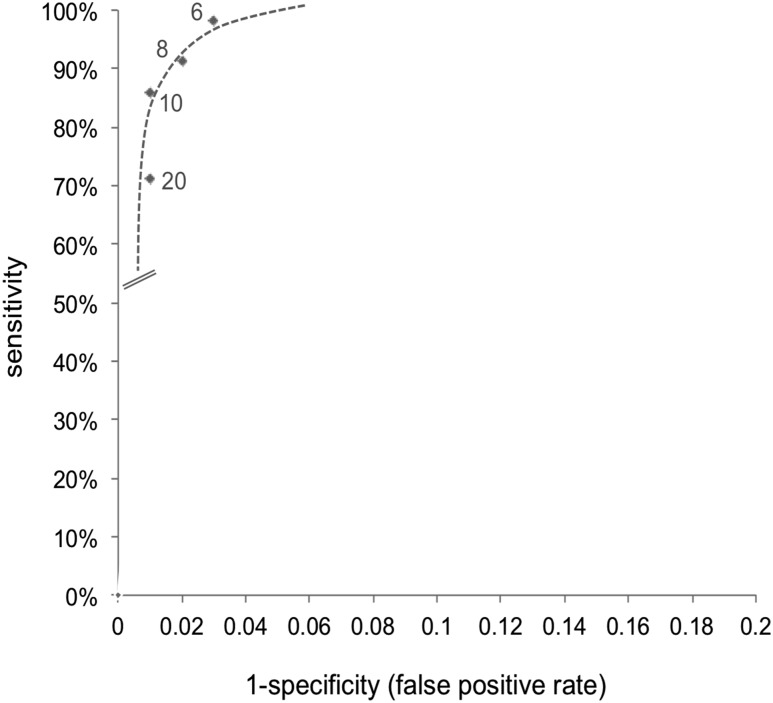
Receiver-operating characteristic curve by English laboratories grouped according to TSH screening thresholds used.

A plot of sensitivity and specificity for each laboratory group (Fig. 2) suggests that the optimal TSH threshold lies between ≥6 and ≥10 mU/L. This was supported by the positive rLR (rLR+) and negative rLR (rLR−) estimated for screening test performance at TSH ≥ 6 mU/L and TSH ≥ 8 mU/L, compared with TSH ≥ 10 mU/L (Table 5). Compared with a TSH threshold ≥10 mU/L, the rLR+ value of ≥8 mU/L was >1 and rLR− was <1. Because the 95% CI for rLR+ included 1, we cannot exclude the possibility that TSH ≥ 8 mU/L does not differ significantly from the current national standard (TSH ≥ 10 mU/L) (15); nevertheless, these results suggest that the negative predictive value for ≥8 mU/L is superior to that for ≥10 mU/L without appreciable reduction in PPV. In contrast, the rLR+ and rLR− were < 1 for TSH ≥ 6 mU/L, suggesting the PPV at TSH ≥ 6 mU/L is inferior to that for ≥10 mU/L. Sensitivity analyses reassigning "probably permanent" CH cases to "not CH" did not change the rLR− values but, compared with TSH ≥ 10 mU/L, the rLR+ for TSH ≥ 8 mU/L decreased to 1.02 (95% CI, 0.76 to 1.37) and the TSH ≥ 6 mU/L remained < 1 [rLR+, 0.66 (95% CI, 0.51 to 0.84)].

**Table 5.** Relative Likelihood Ratios for Screen Thresholds Replacing TSH ≥10 mU/L

Variable	LR+	LR−
TSH ≥6 mU/L as a replacement for TSH ≥10 mU/L^*a*^		
Existing test (TSH ≥10mU/L) (groups 2 and 3; n = 377,914)	5303.00	0.16
Replacement test (TSH ≥6mU/L) (group 1; n = 315,944)	2773.00	0.02
rLR+ (95% CI)	0.52 (0.38, 0.72)	
rLR− (95% CI)	0.11 (0.03, 0.36)	
TSH ≥ 8 mU/L as a replacement for TSH ≥ 10 mU/L^*b*^		
Existing test (TSH ≥10 mU/L) (group 3; n = 252,028)	5632.00	0.16
Replacement test (TSH ≥8 mU/L) (groups 1 and 2; n = 441,830)	4691.00	0.02
rLR+ (95% CI)	1.20 (0.82, 1.75)	
rLR− (95% CI)	0.11 (0.06, 0.20)	

LR, likelihood ratio.

^a^ Screen performance was estimated for all children in laboratory group 1 (TSH ≥ 6 mU/L) and compared with that in all children in laboratory groups 2 and 3 combined (using TSH ≥ 10 mU/L as the screen thresholds).

^b^ Screen performance was estimated for all children in laboratory groups 1 and 2 combined (using TSH ≥ 8 mU/L as the screen thresholds and treating all values below this as negative) and compared with that in all children in laboratory group 3 (TSH ≥10 mU/L).

Had all English laboratories in this study been using TSH ≥ 10 mU/L, 10 children with confirmed permanent CH screened in laboratories using thresholds between TSH ≥ 6 mU/L and <10 mU/L might have been missed (Supplemental Table 3). At a threshold TSH ≥ 8 mU/L, six children would have been screen positive, whereas the remaining four infants had bloodspot TSH values <8 mU/L (one had a congenital syndrome associated with CH, and three had dyshormonogenesis with normal scans).

## Discussion

In a prospective United Kingdom–wide study of CH using active reporting by clinicians and newborn screening laboratories, we found that only two thirds of those with an initial diagnosis of CH following a presumptive positive screening result continued to require thyroxine treatment 3 years later. We estimate that, in England, CH incidence is higher in girls and babies born at <32 weeks’ gestation or those of Asian or Chinese ethnicity, and that the overall incidence is higher than before screening was introduced. Our evaluation of screening program performance demonstrated that the UK program has high sensitivity, specificity, and PPV. Importantly, we have shown that replacing the national recommended threshold of TSH ≥ 10 mU/L with a lower threshold of TSH ≥ 8 mU/L would likely result in improved test performance and identify infants who are currently detected at thresholds below the current recommended threshold, without concomitant increase in false-positive screening results. We found no substantial advantage in test performance using a threshold of TSH ≥ 6 mU/L. Importantly, these thresholds are in relation to the UK screening program, in which the newborn bloodspot is taken at 5 days of age, and therefore these thresholds may not apply to programs that perform screening earlier or later.

We identified a contemporary incidence of CH that is approximately double that reported in the UK population before newborn screening was introduced (16), similar to the increase noted with introduction of screening in other European and North American countries (1, 17). This rise may be related to changes in population demographic characteristics (1, 18) and, in the United Kingdom, ethnic variation in thyroid physiology has been proposed as underlying the growth in screen-detected cases (4). Schoen *et al.* (19) has highlighted variations by sex and ethnicity in the population distribution of mild and severe CH, which may reflect different causes. Because maternal iodine insufficiency leads to raised newborn TSH, higher rates of positive screen results may be partly due to increased prevalence of insufficiency among UK women (5, 6); this merits further investigation.

Lower TSH thresholds may also contribute to the observed increase in CH incidence through increased detection of transient, mild, or subclinical CH; the implications for neurodevelopmental outcomes and need for lifelong treatment are less clear for these children (20, 21). Alm *et al.* (22) reported that children with subclinical CH, defined as raised TSH without other symptoms and signs of CH, had similar neurodevelopmental outcomes to unaffected controls. More recently, Lain *et al.* (23) showed that children with marginally raised newborn TSH results, below the levels indicated for treatment in the Australian program, perform less well educationally than children with treated CH or with negative screen results at lower TSH levels, suggesting the potential for subtle cognitive impairment due to mild CH.

However, lower thresholds lead to significant increases in false-positive rates (20, 24). Korada *et al.* (24) reported a 126% increase in false-positive rate on lowering the TSH threshold from 20 mU/L to 6 mU/L. Furthermore, the investigation of false-positive results increases the costs of screening (25, 26) and can lead to persisting anxiety in parents even after exclusion of CH (27, 28). Children treated for mild or severe CH may experience reduced quality of life (29) compared with unaffected peers, and neurodevelopment may be adversely affected by frequent monitoring, which raises concerns for parents and children (30). The harms of overinvestigation and overtreatment, including continuing treatment lifelong in children for whom CH is not confirmed, are important and should not be ignored when evaluating newborn screening.

As in our study, Ford and LaFranchi found that US newborns who were identified as presumptive positive CH on the first bloodspot were more likely to be girls and to have permanent CH than those who were referred on a repeat test (17). He suggests that the first test may identify infants with prenatal onset of CH due to agenesis or ectopic thyroid, which are more common in girls.

False-negative rates in our study were higher than those reported previously (7); however, these are likely to have been underestimated in previous studies, which used less reliable methods for ascertaining clinically presenting cases. Two studies (31, 32) using multiple sources to capture false-negative cases reported rates of 0.1 and 0.3 per 100,000, respectively, which compares with our rate of 1.1 per 100,000 infants screened. These false-negative or "missed" cases underline the importance of checking thyroid function in older infants who present with clinical manifestations that may indicate hypothyroidism, as inevitably not all cases can be detected by population screening programs even when very low TSH thresholds are used.

Important strengths of our observational study were the complete national coverage of a large population of >800,000 newborns in which all screening laboratories were using AutoDELFIA (Perkin-Elmer) technology, with high ascertainment and follow-up rates. Moreover, the high rate of ascertainment of clinically presenting screen-negative cases permits reliable estimates of screening program performance. Nevertheless, the laboratory source was essential for achieving complete ascertainment because some pediatricians did not report all cases identified as presumptive positive by screening. Pediatricians were more likely to report a screen-positive infant if they started treatment, whereas laboratory staff reported all screen-positive infants regardless of subsequent treatment decisions.

Although differences in screening thresholds between laboratories introduced variability into our estimates of sensitivity and specificity for the screening program as a whole, we were able to take advantage of these to evaluate the influence of bloodspot TSH thresholds on screening performance. Furthermore, using direct population standardization, we ensured that differences among the three laboratory groups, including population ethnicity, were accounted for in our comparative analyses.

Unlike many previous studies, we undertook follow-up to 3 years after initial referral and obtained information about re-evaluation and confirmatory tests throughout this period to inform the final assignment of diagnostic outcome. Had we relied on the diagnosis at onset of therapy, the estimated number of CH cases would have been 16% higher. However, because this was an observational study, clinicians completed questionnaires by using only the data that were routinely available in medical records; therefore, information about the reasons for clinical decisions was limited. We assumed at 3-year follow up that children who continued on a levothyroxine dose of <50 μg per day without re-evaluation or scan confirmation had probably permanent, rather than transient, CH: Should this assumption prove incorrect, this would result in underestimation of transient cases and overestimation of probably permanent CH cases.

Our study demonstrates that in the United Kingdom, 30% of children with a presumptive positive screen continue long-term on thyroxine treatment without a trial off therapy or other confirmation of permanent CH. This underlines the need for a more active approach to re-evaluating CH diagnosis in all children ~2 to 3 years of age to avoid lifelong levothyroxine in children who do not require it.

Analysis of the tradeoff between sensitivity and specificity at screening thresholds of ≥6, ≥8, and ≥10 mU/L suggests that the optimal TSH threshold is likely to be around 8 mU/L for infants screened at 5 days of life. A reduction in screen test thresholds that completely avoids "missed" cases is not feasible and would likely result in more children undergoing unnecessary investigation and treatment of CH. Most children in our study who presented clinically after a negative screen result were identified through investigation of prolonged jaundice or comorbidities.

Existing cost-benefit analyses for the UK screening program for CH are based on preventing severe intellectual disability (33); however, there is no clear evidence that these benefits apply to all types of CH, including children identified at lower screen thresholds. Further investigation of the natural history and benefits of treating mild, transient, or subclinical CH is essential to confirm the benefit or otherwise of extending the current screening program to detect such cases. Further research is essential to understand the characteristics and outcomes for infants with mild or transient CH to offer an effective population screening program that appropriately balances the benefit of early diagnosis against the harms of overinvestigation and overtreatment.

## Supplementary Material

Supplemental Tables

## Abbreviations:

CH: congenital hypothyroidism
IRR: incidence rate ratio
PPV: positive predictive value
rLR: relative likelihood ratio
