# Supplementary material for: Newborn Screening for Primary Congenital Hypothyroidism: Estimating Test Performance at Different TSH Thresholds
Source: J Clin Endocrinol Metab. 2018 Aug 2;103(10):3720–8. doi: 10.1210/jc.2018-00658 (PMC6179177; doi:10.1210/jc.2018-00658)
Supplement: Supplemental Tables [file jc.2018-00658.sd1.docx]

**Supplemental Table S1: Characteristics of 21 children who were clinically detected**

| **Sex** | **Gestation (weeks)** | **Clinical presentation** | **Bloodspot TSH (mU/L)** | **Serum TSH (mU/L)** | **Began treatment** | **Outcome at 3 years** |
| --- | --- | --- | --- | --- | --- | --- |
| F | ≥37 | Jaundice | 3 | 9 | yes | Confirmed permanent CH (trial off therapy) |
| F | ≥37 | Congenital anomaly | 1 | - | yes | Confirmed permanent CH (trial off therapy) |
| F | ≥37 | Suspected before screen; Family history | 7 | 15 | yes | Confirmed permanent CH (high dose thyroxine) |
| M | <32 | Sick neonate | 1 | - | yes | Confirmed permanent CH (trial off therapy) |
| F | 32-37 | Jaundice; Family history | 3 | 13 | yes | Probably permanent CH |
| M | <32 | Jaundice | 1 | 13 | yes | Probably permanent CH |
| M | ≥37 | Jaundice | 5 | 15 | yes | Probably permanent CH |
| F | ≥37 | Jaundice | 5 | 14 | yes | Probably permanent CH |
| M | 32-37 | Congenital anomaly | 4 | 9 | yes | Probably permanent CH |
| M | <32 | Sick neonate | 1 | 29 | yes | Probably permanent CH |
| F | <32 | Congenital anomaly | 4 | 47 | yes | Probably permanent CH |
| M | 32-37 | Suspected before screen; Sick neonate | 8 | 16 | yes | Probably permanent CH |
| F | ≥37 | Suspected before screen; Congenital anomaly | 40 | 100 | yes | Probably permanent CH |
| M | ≥37 | Suspected before screen; Family history | 20 | 98 | yes | Probably permanent CH |
| M | <32 | Congenital anomaly | 2 | 4 | yes | Not CH (trial off therapy) |
| M | 32-37 | Congenital anomaly | - | 14 | yes | Not CH (trial off therapy) |
| F | ≥37 | Jaundice | 4 | 34 | yes | Not CH (trial off therapy) |
| F | ≥37 | Congenital anomaly | - | 16 | yes | Not CH (clinical evaluation) |
| M | ≥37 | Poor growth | - | 55 | yes | Not CH (clinical evaluation) |
| F | 32-37 | Congenital anomaly | - | 16 | yes | Not CH (clinical evaluation) |
| F | <32 | Sick neonate | 14 | 21 | no | Not CH (clinical evaluation) |

**Abbreviations F** female; **M** male; **CH** congenital hypothyroidism

**Supplemental Table S2:** **Screening programme performance at different test cut-offs for a hypothetical population of 100,000 infants**

|  | **Screening performance – comparing cut-offs*** | | | | | | | | |
| --- | --- | --- | --- | --- | --- | --- | --- | --- | --- |
|  | **Group 1****  **Actual population screened n=315944** | | | | **Group 2****  **Actual population screened n=125886** | | | **Group 3****  **Actual population screened n=252028** | |
| **TSH (mU/L)** | ≥6 | ≥8 | ≥10 | >20 | ≥8 | ≥10 | >20 | ≥10 | >20 |
| **Standardised population of screened infants (n)** | **100,000**^†^ | | | | **100,000**^†^ | | | **100,000**^†^ | |
| Expected cases (n) | 57 | | | | 57^#^ | | | 57^##^ | |
| **Positive screen result (n)** | **90** | **63** | **55** | **44** | **75** | **61** | **51** | **67** | **54** |
| True positives (n) | 56 | 49 | 45 | 38 | 55 | 48 | 41 | 48 | 43 |
| False positives (n) | 34 | 14 | 10 | 6 | 20 | 13 | 10 | 19 | 11 |
| **Negative screen result (n)** | **99910** | **99937** | **99945** | **99956** | **99925** | **99939** | **99949** | **99933** | **99946** |
| False negatives (n) | 1 | 8 | 12 | 19 | 2 | 9 | 16 | 9 | 14 |
| True negatives (n) | 99909 | 99929 | 99933 | 99937 | 99923 | 99930 | 99933 | 99924 | 99932 |
| **Detection rate (%)** | **98.2%** | **86.0%** | **79.0%** | **66.7%** | **96.5%** | **84.2%** | **71.9%** | **84.2%** | **75.4%** |
| **PPV (%)** | **62.2%** | **77.8%** | **81.8%** | **86.4%** | **73.3%** | **78.7%** | **80.4%** | **71.6%** | **79.3%** |
| **False positive rate (%)** | **0.03%** | **0.01%** | **0.01%** | **0.01%** | **0.02%** | **0.01%** | **0.01%** | **0.02%** | **0.01%** |

**Notes** *based on 13 newborn screening laboratories in England; screening programme coverage for 2011-2012 was 86%; PPV=positive predictive value;

Group 1 included 6 laboratories of which 1 used a threshold of ≥5mU/L and 5 used a threshold of ≥6mU/L; Group 2 included 3 laboratories, all using a threshold of ≥8mU/L; Group 3 included 4 laboratories, all using a threshold of ≥10mU/L. As exact values for TSH are not provided for negative screen results, these were reported only as ‘negative’, therefore estimates can only be made of screening performance at a cut-off TSH ≥6mU/L for Group 1 (and not for Groups 2 and 3).

** The percentage who were of white ethnicity were 68% in Group 1, 79% in Group 2 and 70% in Group 3

^†^ 100,000 live births **directly standardised**^15^ using the actual English population profile (by sex, ethnicity and gestation) for 2011-2012

^#^ 1 additional false negative to account for case detected at cut-off of 6 in Group 1

^##^ 2 additional false negatives to account for cases detected at cut-off of 6 in Group 1

Total number of babies screened (Table S2) is greater (n=110 [0.01% of 800,000) than the total English live births reported in Table 3 as the data sources varied. This is because the denominator in Table 3 was from monthly figures provided by the Office for National Statistics for *live births* occurring in England, while the ‘screened population’ denominator in Table S2 was provided by the screening programme from annual reporting of babies *screened* in England. The minor discrepancy may be due to several reasons, including some births occurring outside England but being screened in England in border areas, or inaccuracies in national reporting systems. The relevant denominator was therefore used for each table.

**Supplemental Table S3: Children who would have been missed at the national screening cut-off TSH≥10mU/L**

|  | **Gestation** | **Bloodspot TSH (mU/L)** | **Serum TSH (mU/L)** | **SerumT4 (pmol/L)** | **Confounding factors** | **Scan** | **CH confirmation** |
| --- | --- | --- | --- | --- | --- | --- | --- |
| **Children who would be detected at TSH≥6mU/L but not at TSH≥8mU/L** | | | | | | | |
| A | ≥37 weeks | 6.3 | 50.5 | 13.7 | Congenital syndrome | Abnormal | Agenesis (scan) |
| B | ≥37 weeks | 6.8 | 16.4 | 17.0 | None | Normal | Trial-off therapy |
| C | ≥37 weeks | 7.8 | 22.0 | NK | None | Abnormal | Trial-off therapy |
| D | ≥37 weeks | 7.0 | 64.2 | 14.4 | None | Abnormal | Trial-off therapy |
| **Children who would be detected at TSH≥8mU/L but not at TSH≥10mU/L** | | | | | | | |
| E | <32 weeks | 8.0 | NK | NK | None | Normal | Trial-off therapy |
| F | ≥37 weeks | 8.0 | 51.8 | 19.0 | None | Abnormal | High dose thyroxine |
| G | <32 weeks | 9.3 | NK | 3.6 | None | Normal | Trial-off therapy |
| H | ≥37 weeks | 9.3 | 20.3 | 12.4 | None | Abnormal | Trial-off therapy |
| J | 32-37 weeks | 9.9 | 17.6 | NK | None | No scan | High dose thyroxine |
| K | ≥37 weeks | 9.9 | 16.0 | 13.0 | None | Abnormal | High dose thyroxine |

**Notes** Total screened population (Groups 1 and 2) of 441830 newborns. TSH and T4 levels were those recorded at initial clinical referral after a positive screening result. Abnormal scans showed avid or poor uptake and were suggestive of dyshormonogenesis, except for the scan confirming agenesis. **Abbreviations** **TSH** thyroid stimulating hormone; **CH** congenital hypothyroidism; **NK** not known (information not found in medical records)
